# Kentucky Shower of Flesh

**Published:** 1876-07

**Authors:** 


### KENTUCKY SHOWER OF FLESH.

The so-called shower of flesh in Kentucky is made the subject of a communication to *The Sanitarian* for May, 1876, by Leopold Brandeis of Brooklyn. The writer said that in 1537, while Paracelsus was engaged in the production of his "elixir of life," he came across a very strange looking vegetable mass, to which he gave the name of "nostoc."

The want of rapid transportation, combined with the perishable nature of the subtances fallen, have hitherto prevented a complete and exhaustive examination. The specimens of the "Kentucky shower," however, reached this city well preserved in glycerin, and it has been comparatively easy to identify the substance, and to fix its status. The "Kentucky wonder" is nothing more or less than the nostoc of the old alchemist. The nostoc belongs to the confervæ; it consists of translucent, gelatinous bodies joined together by thread-like tubes of seed bearers. There are about fifty species of this singular plant classified; two or three kinds have even been found in a fossil state. Like other confervæ, the nostoc propagates by self-division as well as by seeds or spores. When these spores work their way out of the gelatinous envelope they may be wafted by the winds here and there, and they may be carried great distances. Wherever they may fall, and find congenial soil, namely, dampness or recent rain, they will thrive and spread very rapidly, and many cases are recorded where they have covered miles o ground in a very few hours with long strings of nostoc.

On account of the rapidity of growth, people almost everywhere faithfully believe the nostoc to fall from the clouds, and ascribe to it many mysterious virtues. The plant is not confined to any special locality or to any climate; sown by the whirlwind, carried by a current of air, in need of moisture only for existence and support, it thrives everywhere. Icebergs afloat amid ocean have been found covered with it. In New Zealand it is found in large masses of quaking jelly, several feet in circumference, and covering miles of damp soil; and in our own country it may be found in damp woods, on meadows and on marshy, or even gravelly bottoms.

All the nostocs are composed of a semi-liquid cellulose and vegetable proteine. The edible nostoc is highly valued in China, where it forms an esseetial ingredient of the edible bird-nest soup. The flesh that was supposed to have fallen from the clouds in Kentucky, is the flesh-colored nostoc (*N. curneum* of the botanist); the flavor of it approaches frog, or spring chicken legs, and it is greedily devoured by almost all domestic animals. Such supposed "showers" are not rare, and are in entire harmony with natural laws. In the East Indies the same nostoc is used as an application in ulcers and scrofulous disease, while every nation in the East considers it nourishing and palatable. and uses it even for food when dried by sun-heat.—*Boston Med, Surg. Jour.*

